# Psycho-social factors associated with high depressive symptomatology in female adolescents and gender difference in adolescent depression: an epidemiological survey in China’s Hubei Province

**DOI:** 10.1186/s12888-021-03165-7

**Published:** 2021-03-26

**Authors:** Wenzhe Sun, Junhua Mei, Yanyan Wang, Xin Zhao, Zhou Zhu, Chenyan Zhang, Chensheng Pan, Guo Li, Yuxi Chen, Jinfeng Miao, Yan Lan, Xiuli Qiu, Yi Xu

**Affiliations:** 1 Department of Neurology, Tongji Hospital, Tongji Medical College, Huazhong University of Science and Technology, No.1095 Jiefang Avenue, Wuhan, 430030 China; 2 Department of Neurology, Wuhan First Hospital, No.215 Zhongshan Avenue, Wuhan, 430030 Hubei China; 3 The Solomon H. Snyder Department of Neuroscience, Johns Hopkins University School of Medicine, Baltimore, MD 21205 USA; 4 Department of Plastic surgery, Tongji Hospital, Tongji Medical College, Huazhong University of Science and Technology, No.1095 Jiefang Avenue, Wuhan, 430030 China

**Keywords:** Female, Adolescents, Gender difference, Depressive symptomatology, Psycho-social factors

## Abstract

**Background:**

Exploring etiological clues to adolescent depression, especially in female adolescents, might be helpful to improve the social environment of female adolescents. The aim at this study is to explore psycho-social factors of female adolescents with high depressive symptomatology and gender differences in depressive symptoms among Chinese adolescents.

**Method:**

We examined 4100 adolescents from Wuhan city and Jianli county via a cross-sectional study. Depressive symptomatology was screened through the Chinese version of Center for Epidemiology Studies Depression Scale. Multivariate logistic regression was performed to explore the factors related to high depressive symptomatology in female and male adolescents, respectively.

**Results:**

The prevalence of high depressive symptomatology in female and male were 38.9 and 30.2% respectively. The psycho-social factors of high depressive symptomatology in female adolescents were age (Adjusted odds ratio [aOR] = 1.201, 95% confidence interval [CI], 1.076 ~ 1.341), single parent family (aOR = 2.004, 95%CI, 1.448 ~ 2.772) and fathers’ education level (compared to primary school and below, [Junior middle school, aOR = 0.641, 95%CI, 0.439 ~ 0.934; Senior middle school, aOR = 0.603, 95%CI, 0.410 ~ 0.888; College degree and above, aOR = 0.639, 95%CI, 0.437 ~ 0.936]).

**Conclusion:**

Fathers’ education level was associated with high depressive symptomatology in female adolescents. Female adolescents whose father with primary school education or below deserves more attention. Further epidemiologic researches need to be conducted to explore the different risk factors between female and male adolescents in China.

## Introduction

For the past few years, depression have become a major public health problem worldwide [1]. According to the World Health Organization (WHO), depression will be the leading cause of disease burden by 2030 [2]. In China, the new update from the National Health and Health Commission (2018) shows that about 30 million children and adolescents under the age of 17 are suffering from psychological disorders in China [3], while psychological disorders such as depression, anxiety and bipolar disorder have been neglected for a long time in China. Given that quite a few depression diagnoses have their initial origins in adolescence, it is important to identify depression and prevent the establishment of maladaptive cognitive or behavioral patterns among early adolescence [4–6].

The sociodemographic characteristics of China may play a prominent role in the occurrence of depressive symptoms in Chinese adolescents, since adolescents are more vulnerable to the surrounding environment and the individual psychological factors [7–10]. The situation of female adolescents in China is even more difficult. Gender factors have already been widely discussed and a considerable number of studies suggested that females, both adults and adolescents, have higher risk of depression than males [11, 12]. Furthermore, owing to entrenched traditional social roles, son preference has always been a common problem in China. In recent years, early marriage and procreation rate of women rise again, although they dropped between 1990 and 2015 [13], which suggested that the educational inequities in female adolescents of China has recently exacerbated. However, few researchers have explored the different sociodemographic related factors between female adolescents and male adolescents.

Hence, it’s necessary to conduct an exhaustive study about gender difference of psycho-social factors in China to provide clues for early intervention of adolescent depression. Furthermore, Hubei province, the place targeted in this study, had the highest rates of suicide in China [14], so it is appropriate for depression research. The objective of this study is to explore psycho-social factors of female adolescents with high depressive symptomatology and gender differences in depressive symptoms among Chinese adolescents.

## Method

### Participants

This epidemiological survey was conducted from November 2018 to February 2019 in Wuhan city and Jianli country of Hubei province, to define the prevalence and associated factors for high depressive symptomatology in female and male adolescents. This cross-sectional study was approved by our Institutional Review Board and participants were recruited for the project with approval from the school officials, and with approval from their guardians in written consent with the assistance of teachers. Three junior middle schools of Wuhan city and four junior middle schools of Jianli county were selected with stratified-cluster sampling method. The inclusion criteria for this study were: (1) adolescents in grades 7 to 9; (2) adolescents who were willing to take part in the survey. Individuals were excluded if any of the following criteria was met: (1) adolescents were unwilling to take part in the survey; (2) Adolescents did not complete the questionnaire. Eventually, 4,122 students were approached, 22 students declined to participate or incomplete the questionnaires, 4,100 junior high school students covering three grades aged from 11 to 16 years old were involved in analysis.

### Data collection

The investigation was organized and coordinated by Department of Neurology, Tongji Hospital, Tongji Medical College, Huazhong University of Science and Technology. The investigators who conducted the questionnaire survey were trained uniformly. The survey was conducted through teachers before COVID, all data were collected via a paper demographic survey handed out in class by the adolescents themselves. The senior investigators checked the collected questionnaires daily to perform quality control. Data were entered double-blindly into the database by two different researchers using Epidata 3.0 to guarantee accuracy. All the data were collected through paper questionnaires, each of which contained an informed consent form filled out by the subject’s parents or guardians.

### Outcomes

Depressive symptomatology was screened through the Chinese version of Center for Epidemiology Studies Depression Scale (CES-D Scale). A score of 20 and above indicated high depressive symptomatology [15–19]. CES-D has been widely used in Chinese adolescents, with good reliability and validity [20, 21]. The Cronbach’s α coefficients of the CES-D Scale in this study were 0.87.

### Variables

The demographic characteristics were collected, consisting of rural area students, age, gender, boarding status, class status, household characteristics, single parent family, current caregivers, sibling child status, left behind types, father’s and mother’s education level, physical activity (PA), birth order and having siblings or not. The children are considered as the left-behind children (LBC) if his/her mother or father has left the local place for a job over the past 5 months. The left behind children were further divided into three subgroups: father absent, mother absent and both absent. Physical activity (PA) were assessed by Global Physical Activity Questionnaire, which were defined as up to the WHO physical activity standards that children and youth aged 5–17 years old should accumulate at least 60 min of moderate- to vigorous-intensity physical activity daily, most of the daily physical activity should be aerobic, and vigorous-intensity activities should be incorporated, including those that strengthen muscle and bone, at least 3 times per week.

### Statistical analyses

Statistical analyses to identify related factors were performed using SPSS software version 22.0 (Statistical Package for the Social Sciences) for Windows (SPSS, Chicago, IL). Continuous data were presented as the medians and interquartile ranges (IQR) and compared using Mann Whitney U test. Categorical variables are presented as frequencies (proportions) and compared with Chi-square test or Fisher’s exact test. The univariate analyses in male and female subgroups were using univariate logistic regression. Variables with a *P* value less than 0.1 in univariate analyses were subjected to multivariable logistic regression analysis. Multivariate logistic regression was performed to explore the factors related to high depressive symptomatology and calculate the adjusted odds ratios (ORs) and 95% confidence intervals (CIs). All *P*-values were two-sided, and *P* <  0.05 was considered statistically significant.

## Results

### Sample characteristics

Table 1 presents comparison of social characteristics of the adolescents with high depressive symptomatology and low depressive symptomatology and factors associated with high depressive symptomatology. In this sample, the age distribution was 11 to 16 years. The total prevalence of high depressive symptomatology was 34.0%. The adolescents with high depressive symptomatology were older (*p* <  0.001), more female (*p* <  0.001), more key class students (*p* = 0.004), getting less physical activity (*p* <  0.001) and more likely from single parent family (*p* <  0.001). All variables with *p* <  0.1 in univariate analysis were included in multivariable logistic regression models for adjustment (Table 1). After multivariate logistic regression analysis, we found that age (aOR = 1.150, 95%CI, 1.062 ~ 1.260, *p* = 0.001), female (aOR = 1.444, 95%CI, 1.260 ~ 1.655, *p* <  0.001), single parent family (aOR = 1.870, 95%CI, 1.524 ~ 2.294, *p* <  0.001) and physical activity (aOR = 0.678, 95%CI, 0.572 ~ 0.804, *p* <  0.001) were independently associated with high depressive symptomatology.

**Table 1:** Related factors of adolescent high depressive symptomatology in Hubei province

Correlates	Univariate analysis			Multivariate analysis	
	High depressive symptomatology (*N* = 1393)	Low Depressive symptomatology (*N* = 2707)	*P*	Adjusted OR (95%CI)	*P*
Rural areas, N (%)	626 (44.9)	1298 (47.9)	0.067	1.065 (0.900 ~ 1.259)	0.464
Age, y (IQR)	14 (13 ~ 14)	13 (13 ~ 14)	< 0.001*	1.150 (1.062 ~ 1.260)	0.001*
Female, N (%)	690 (49.5)	1084 (40.0)	< 0.001*	1.444 (1.260 ~ 1.655)	< 0.001*
Boarding status, N (%)	443 (31.8)	894 (33.3)	0.429		
Key class, N (%)	645 (46.3)	1127 (41.6)	0.004*	1.128 (0.980 ~ 1.299)	0.093
Three generational household, N (%)	1070 (76.8)	2096 (77.4)	0.656		
Single parent family, N (%)	221 (14.4)	228 (8.4)	< 0.001*	1.870 (1.524 ~ 2.294)	< 0.001*
Current caregivers, N (%)			0.797		
Parents	1160 (83.3)	2233 (82.5)			
Grandparents	203 (14.6)	416 (15.4)			
Other relatives	30 (2.2)	58 (2.1)			
Sibling children, N (%)	769 (55.2)	1512 (55.9)	0.691		
Left behind types, N (%)			0.109		
Un-left-behind	933 (67.0)	1896 (70.0)			
Father absent	94 (6.7)	149 (5.5)			
Mother absent	42 (3.0)	62 (2.30			
Both absent	324 (23.3)	600 (22.2)			
Father’s education level, N (%)			0.265		
Primary School and Below	140 (10.1)	236 (8.7)			
Junior middle school	427 (32.1)	915 (33.8)			
Senior middle school	362 (26.0)	738 (27.3)			
College degree and above	444 (31.9)	818 (30.2)			
Mother’s education level, N (%)			0.665		
Primary School and Below	264 (19.0)	514 (19.0)			
Junior middle school	387 (27.8)	800 (29.6)			
Senior middle school	352 (25.3)	662 (24.5)			
College degree and above	390 (28.0)	731 (27.0)			
Physical activity, N (%)	242 (17.4)	650 (24.0)	< 0.001*	0.678 (0.572 ~ 0.804)	< 0.001*
Birth order, N (%)			0.414		
Only children	624 (44.8)	1195 (44.1)			
Eldest children	375 (26.9)	683 (25.2)			
Middle children	116 (8.3)	250 (9.2)			
Youngest children	278 (20.0)	579 (21.4)			
Having younger brother(s), N (%)	373 (26.8)	593 (25.6)	0.416		
Having younger sister(s), N (%)	209 (15.0)	384 (14.2)	0.481		
Having elder brother(s), N (%)	205 (14.7)	395 (14.6)	0.915		
Having elder sister(s), N (%)	268 (19.2)	583 (21.5)	0.086	1.014 (0.856 ~ 1.202)	0.869

*OR* odds ratio, *CI* confidence interval

*Statistically significant at *p* <  0.05 level, two-sided

### Factors associated with high depressive symptomatology among female and male samples

We also compared the psycho-social characteristics of the female and male adolescents (Table 2). The prevalence of high depressive symptomatology in female and male were 38.9 and 30.2% respectively. The adolescents in female group were more key class students (*p* <  0.001), more raised by parents (*p* = 0.001), with higher fathers’ and mothers’ education background (*p* <  0.001), more likely to be the oldest children (*p* <  0.001) and have younger brother in family (*p* <  0.001). On the other hand, adolescents in female group were less rural students (*p* <  0.001), less from single parent family (*p* = 0.034), less likely to have physical activity (*p* <  0.001), less likely to live in school (*p* <  0.001) and have older sister in family (*p* <  0.001).

**Table 2:** Characteristics of male and female adolescents

Variables	Male (*N* = 2326)	Female (*N* = 1774)	*P*
Rural areas, N (%)	1193 (51.3)	731 (41.2)	< 0.001*
Age, y (IQR)	13 (13 ~ 14)	13 (13 ~ 14)	0.707
Boarding status, N (%)	862 (37.1)	475 (26.8)	< 0.001*
Key class, N (%)	931 (40.0)	841 (47.4)	< 0.001*
Three generational household, N (%)	1795 (77.2)	1371 (77.3)	0.933
Single parent family, N (%)	264 (11.3)	165 (9.3)	0.034*
Current caregivers, N (%)			0.001*
Parents	1883 (81.0)	1510 (85.1)	
Grandparents	381 (16.4)	238 (13.4)	
Other relatives	62 (2.7)	26 (1.5)	
Sibling children, N (%)	1280 (55.0)	1001 (56.4)	0.373
Left behind types, N (%)			0.151
Un-left-behind	1576 (67.8)	1253 (70.6)	
Father absent	139 (6.0)	104 (5.9)	
Mother absent	57 (2.9)	47 (2.6)	
Both absent	554 (23.8)	370 (20.9)	
Father’s education level, N (%)			< 0.001*
Primary School and Below	233 (10.0)	143 (8.1)	
Junior middle school	836 (35.9)	526 (29.7)	
Senior middle school	608 (26.1)	492 (27.7)	
College degree and above	649 (27.9)	613 (34.6)	
Mother’s education level, N (%)			< 0.001*
Primary School and Below	486 (20.9)	292 (16.5)	
Junior middle school	707 (30.4)	480 (27.1)	
Senior middle school	552 (23.7)	462 (26.0)	
College degree and above	581 (25.0)	540 (30.4)	
Physical activity, N, (%)	616 (26.5)	276 (15.6)	< 0.001*
Birth order, N, (%)			< 0.001
Only children	1046 (45.0)	773 (43.6)	
Eldest children	444 (19.1)	614 (31.6)	
Middle children	235 (10.1)	131 (7.4)	
Youngest children	601 (25.8)	256 (14.4)	
Having younger brother(s), N, (%)	466 (20.0)	600 (33.8)	< 0.001*
Having younger sister(s), N, (%)	358 (15.4)	235 (13.2)	0.053
Having elder brother(s), N, (%)	355 (15.3)	254 (13.8)	0.193
Having elder sister(s), N, (%)	640 (27.5)	211 (11.9)	< 0.001*
High depressive symptomatology, N, (%)	703 (30.2)	690 (38.9)	< 0.001

*Statistically significant at *p* <  0.05 level, two-sided

Furthermore, logistic regression was used to identify factors associated with high depressive symptomatology in female and male adolescents respectively (Table 3&4). The related factors of high depressive symptomatology in female adolescents were age (aOR = 1.201 95% CI, 1.076 ~ 1.341), single parent family (aOR = 2.004, 95%CI, 1.448 ~ 2.772) and fathers’ education level (compared to primary school and below, [Junior middle school, aOR = 0.641, 95%CI, 0.439 ~ 0.934; Senior middle school, aOR = 0.603, 95%CI, 0.410 ~ 0.888; College degree and above, aOR = 0.639, 95%CI, 0.437 ~ 0.936]) (Table 4). The factors independently associated with high depressive symptomatology in male adolescents were age, single parent family and physical activity.

**Table 3:** Univariate and multivariate logistic regression analysis for high depressive symptomatology in male adolescents

Correlates	Univariate analysis		Multivariate analysis	
	*P*	Crude OR (95%CI)	*P*	Adjusted OR (95%CI)
Rural areas	0.439	0.932 (0.781 ~ 1.113)		
Age	0.009*	1.129 (1.031 ~ 1.236)	0.009*	1.145 (1.034 ~ 1.266)
Boarding status	0.328	1.095 (0.913 ~ 1.315)		
Key class	0.030*	1.220 (1.020 ~ 1.461)	0.058	1.206 (0.994 ~ 1.464)
Three generational household	0.627	0.949 (0.770 ~ 1.171)		
Single parent family	< 0.001*	1.801 (1.386 ~ 2.342)	< 0.001*	1.734 (1.327 ~ 2.265)
Current caregivers	0.783			
Parents		1.00		
Grandparents	0.546	0.928 (0.728 ~ 1.182)		
Other relatives	0.758	1.089 (0.634 ~ 1.871)		
Sibling children	0.938	0.993 (0.831 ~ 1.186		
Left behind types	0.072		0.071	
Un-left-behind		1.00		1.00
Father absent	0.035*	1.473 (1.027 ~ 2.112)	0.053	1.436 (0.996 ~ 2.071)
Mother absent	0.115	1.549 (0.899 ~ 2.669)	0.181	1.462 (0.838 ~ 2.550)
Both absent	0.260	1.128 (0.915 ~ 1.392)	0.077	1.225 (0.978 ~ 1.534)
Father’s education level	0.883			
Primary School and Below		1.00		
Junior middle school	0.824	0.965 (0.704 ~ 1.322)		
Senior middle school	0.611	0.918 (0.661 ~ 1.275)		
College degree and above	0.950	1.011 (0.731 ~ 1.397)		
Mother’s education level	0.928			
Primary School and Below		1.00		
Junior middle school	0.942	0.991 (0.770 ~ 1.275)		
Senior middle school	0.907	0.984 (0.754 ~ 1.285)		
College degree and above	0.651	1.062 (0.818 ~ 1.380)		
Physical activity	< 0.001*	0.666 (0.539 ~ 0.822)	< 0.001*	0.632 (0.509 ~ 0.783)
Birth order	964			
Only children		1.00		
Eldest children	0.692	0.952 (0.747 ~ 1.214)		
Middle children	0.978	0.996 (0.732 ~ 1.355)		
Youngest children	0.840	1.023 (0.823 ~ 1.271)		
Having younger brother(s)	0.749	0.964 (0.772 ~ 1.204)		
Having younger sister(s)	0.635	1.061 (0.832 ~ 1.353)		
Having elder brother(s)	0.734	1.043 (0.817 ~ 1.333)		
Having elder sister(s)	0.954	1.006 (0.825 ~ 1.226)		

*OR* odds ratio, *CI* confidence interval

*Statistically significant at *p* <  0.05 level, two-sided

**Table 4:** Univariate and multivariate logistic regression analysis for high depressive symptomatology in female adolescents

Correlates	Univariate analysis		Multivariate analysis	
	*P*	Crude OR (95%CI)	*P*	Adjusted OR (95%CI)
Rural areas	0.307	0.904 (0.744 ~ 1.098)		
Age	0.003*	1.172 (1.057 ~ 1.300)	0.001*	1.201 (1.076 ~ 1.341)
Boarding status	0.161	0.856 (0.689 ~ 1.064)		
Key class	0.209	1.130 (0.934 ~ 1.369)		
Three generational household	0.884	0.983 (0.783 ~ 1.235)		
Single parent family	< 0.001*	2.018 (1.461 ~ 2.786)	< 0.001*	2.004 (1.448 ~ 2.772)
Current caregivers	0.997			
Parents		1.00		
Grandparents	0.953	1.009 (0.762 ~ 1.335)		
Other relatives	0.966	1.089 (0.634 ~ 1.871)		
Sibling children	0.533	0.941 (0.776 ~ 1.140)		
Left behind types	0.783			
Un-left-behind		1.00		
Father absent	0.652	1.098 (0.730 ~ 1.652)		
Mother absent	0.542	1.201 (0.666 ~ 2.165)		
Both absent	0.407	1.105 (0.872 ~ 1.401)		
Father’s education level	0.131		0.74	
Primary School and Below		1.00		1.00
Junior middle school	0.023*	0.650 (0.448 ~ 0.943)	0.021*	0.641 (0.439 ~ 0.934)
Senior middle school	0.033*	0.665 (0.457 ~ 0.968)	0.010*	0.603 (0.410 ~ 0.888)
College degree and above	0.078	0.719 (0.499 ~ 1.037)	0.021*	0.639 (0.437 ~ 0.936)
Mother’s education level	0.584			
Primary School and Below		1.00		
Junior middle school	0.299	0.854 (0.633 ~ 1.151)		
Senior middle school	0.939	1.012 (0.751 ~ 1.364)		
College degree and above	0.594	0.924 (0.691 ~ 1.236)		
Physical activity	< 0.054*	0.767 (0.585 ~ 1.005)	0.073	0.778 (0.592 ~ 1.023)
Birth order	0.508			
Only children		1.00		
Eldest children	0.944	1.008 (0.812 ~ 1.251)		
Middle children	0.245	0.794 (0.539 ~ 1.171)		
Youngest children	0.335	0.866 (0.646 ~ 1.160)		
Having younger brother(s)	0.867	1.017 (0.832 ~ 1.245)		
Having younger sister(s)	0.422	1.121 (0.848 ~ 1.483)		
Having elder brother(s)	0.967	0.994 (0.754 ~ 1.311)		
Having elder sister(s)	0.225	0.830 (0.615 ~ 1.121)		

*OR* odds ratio, *CI* confidence interval

*Statistically significant at *p* < 0.05 level, two-sided

## Discussion

In this study, we aimed to explore psycho-social factors of female adolescents with high depressive symptomatology and gender differences in depressive symptoms among Hubei province. The total prevalence of this study was 34.0%, it’s similar to other areas in East Asia, including Shenzhen (34.7%), Taiwan (30.2%) and Korea (31.4%) [22–24].

It is a unique phenomenon in China, especially in China’s urban areas, that middle school students were divided into key classes and non-key classes according to their academic achievement [25]. Key classes mean “better students”, “better teachers” and more high-quality educational resources, and adolescents in non-key class may experience discrimination. In this study, we found that compared to low depressive symptomatology, high depressive symptomatology group have more key class students (46.3% VS. 41.6%). However, after adjusting for confounding variables, key class was not a significantly associated factor for presenting with high depressive symptomatology.

New research from the UK is suggesting that higher birth order (later born) children were at increased risk of suicide attempts and psychiatric disorders in adolescence [26] and sibling bullying may be a significant driver for this phenomenon [27, 28]. Contrary to previous survey, we mentioned that birth order was not significantly associated with high depressive symptomatology in both male and female adolescents. We suspect it because parents in China tend to favor the younger children, especially their youngest sons. The results of the present study also support this idea, we can see that male adolescents were obviously more than female adolescents among these youngest children (*N* = 857, 70.1% VS. 29.9%).

Recent years, the physical psychological problems of left-behind children in China have aroused extensive concerns. Left-behind children have more mental and behavioral problems compared with general population of children [29, 30]. Meanwhile, some studies also present different results [31]. In China, the long-term impacts of traditional concepts lead parents to focus almost exclusively on their children’s academic performance rather than on psychological well-being [32]. Adolescents living with parents might suffering physical and verbal punishment when they didn’t study hard, which increased the risk of high depressive symptomatology. Some researchers have also found that adolescents whose father came back home more frequently displayed poorer health-related quality of life [33]. Thus, the current view on relationship between left-behind and depressive symptoms remains controversial. In this study, left behind status were not significantly related to high depressive symptomatology in both mela and female adolescents.

This study shown that the prevalence of high depressive symptomatology in female adolescents was significantly higher than male adolescents. Further subgroup analysis revealed that age, single parent family and fathers’ education background were associated with high depressive symptomatology in female adolescents, fathers’ education background was only associated with high depressive symptomatology in female adolescents, but not with male adolescents.

**Fig. 1:**
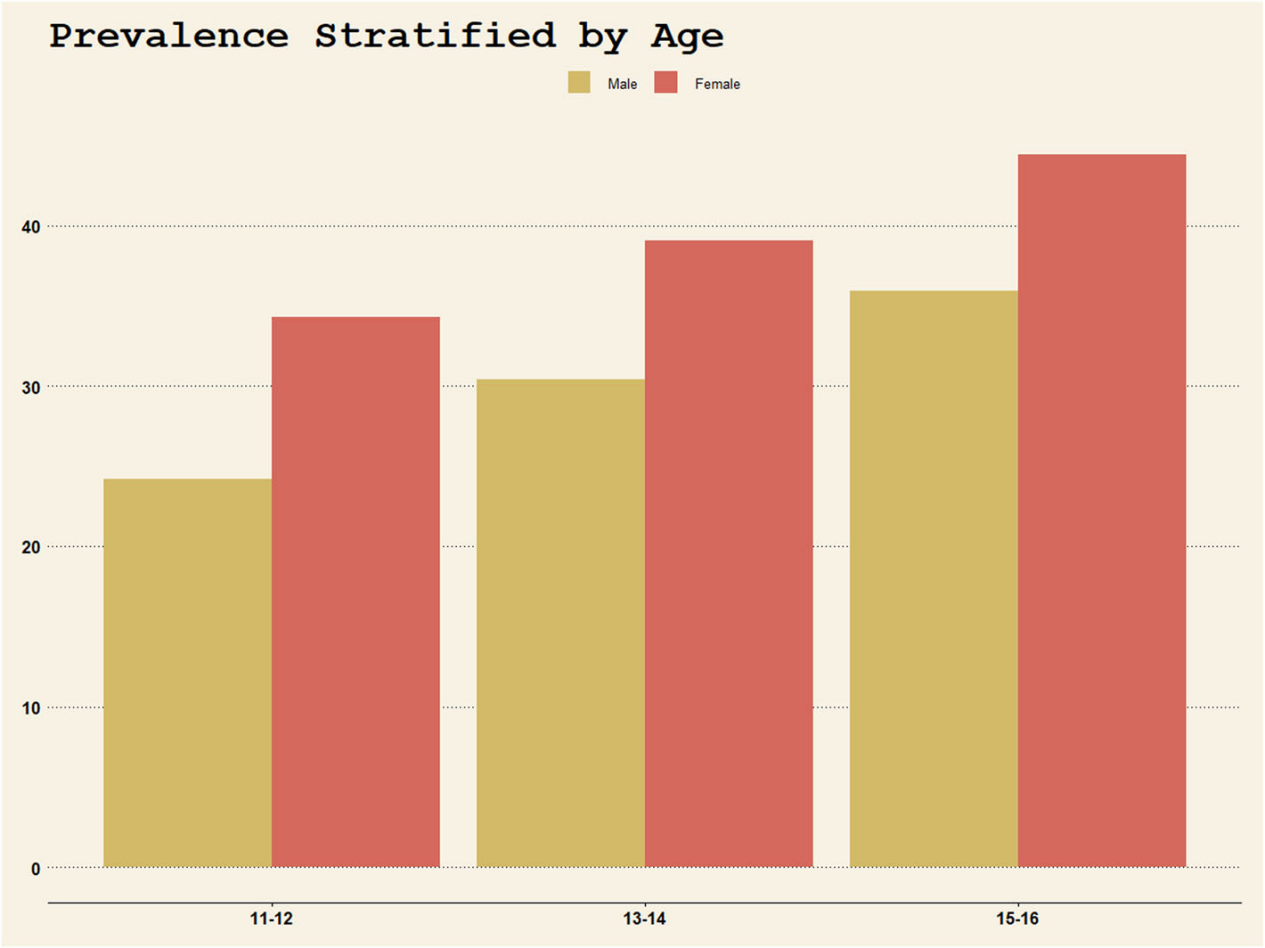
Prevalence stratified by age

Previous research has suggested that females, whether adults or adolescents, were more likely to suffer depression, and this gender difference emerges from adolescence [34]. As shown in Fig. 1, in this study, the prevalence increased with age in both female adolescents and male adolescents (female 34.3% vs. male 24.2% in 11–12 group, 39.1% vs. 30.4% in 13–14 group, 44.4% vs. 35.9% in 15–16 group, respectively), and the gender difference existed in each age group. Previous studies suggested that the gender difference of depression in adolescents emerges between ages 12 and 15, while females experience increases in depression symptoms in early adolescence and males experience increases in depression symptoms in later adolescence [35–38]. A recent longitudinal study indicated that girls’ diagnostic rates of depression increased from ages 11 through 14, whereas boys’ diagnostic rates of depression increased from ages 15 through 18 [39]. However, all these researches were conducted in United States. The national adolescent sample in China showed that prevalence of depressive symptoms in both boys and girls had increased from 7th to 9th grade [40]. This may be due to the cultural differences between China and United States. Asian cultures often value modesty and self-effacement as cultural virtues and inhibit the expression of positive emotion [41, 42], which would also lead to differences in prevalence between male and female adolescents. A cross-cultural comparative study found that after controlling for level of income, age, education, and previous chemotherapy, there was a significant cultural difference in the positive affect subscale between Chinese and US female [43]. These results are consistent with other cross-cultural comparisons of CES-D scores from healthy community Asian populations including South Koreans, Japanese, and Chinese, which have reported that scores on positive affect components are lower, and can lead to higher depressive symptom scores [41, 42, 44–46].

**Fig. 2:**
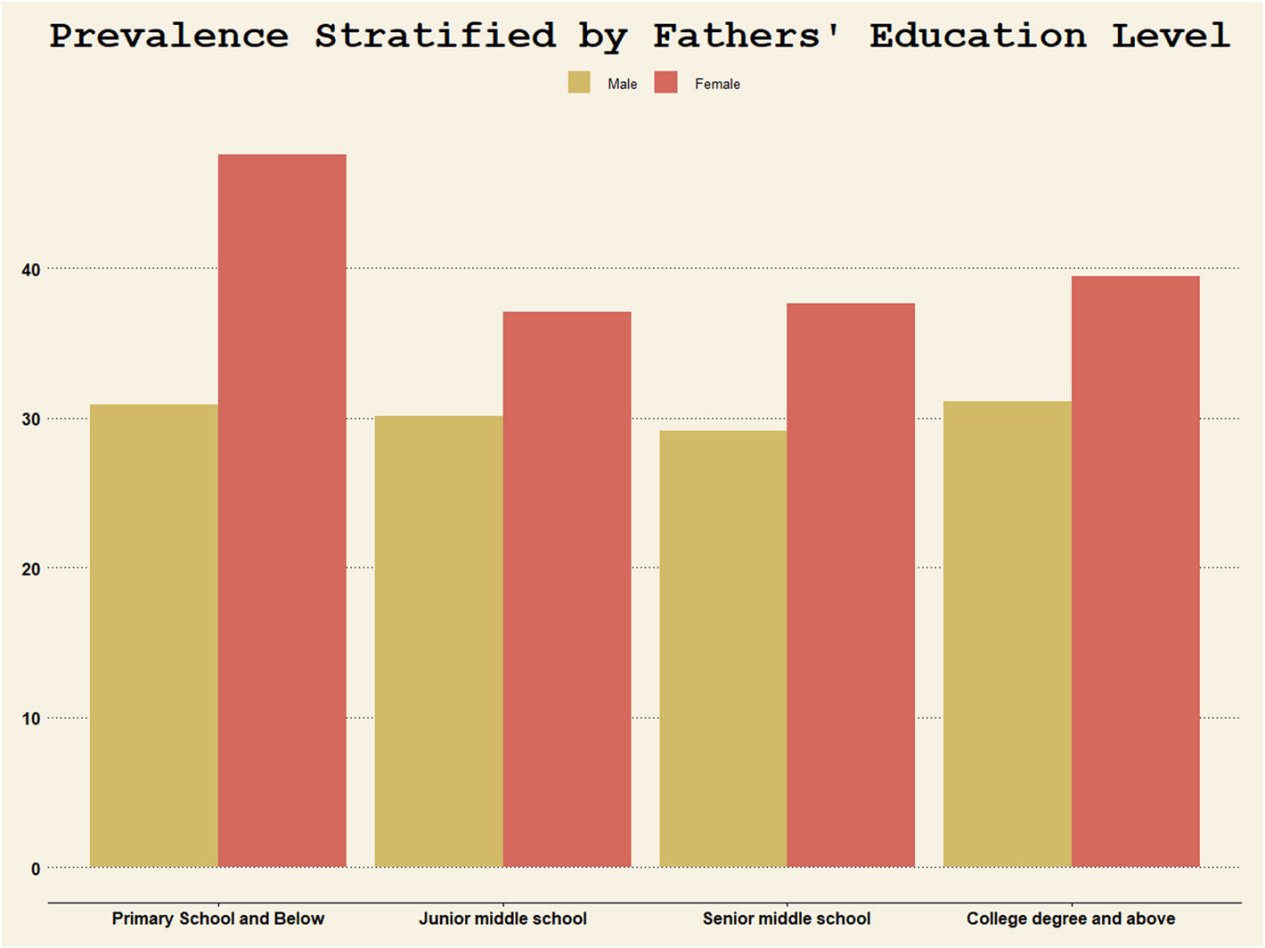
Prevalence stratified by fathers’ education level

Subgroup analysis shows that female adolescents whose father with primary school education or below were more susceptible to high depressive symptomatology (Table 4). From Fig. 2 we can see that prevalence of high depressive symptomatology in girls whose father with primary school education or below was much higher than any other girls, whereas prevalence of high depressive symptomatology in male adolescents has remained relatively unaffected by fathers’ education level.

Traditional preferences for sons are deep-rooted in rural areas of China, especially in low educated communities [47–50]. According to the Global Gender Gap Report 2018, China ranked dead last among 149 countries in terms of “sex ratio at birth”. This is the result of many factors, such as the Confucian cultural tradition, the socioeconomic system, and gender ideology. In the context of traditional patriarchal, patrilineal and patriarchal systems, sons are considered to have unique value, as they inherit the family name and property and represent an economic value premium to the family and parents [48, 50–52]. Under the one-child policy in China, a family with strong preference for sons may be trying to conduct gender selection [49]. Since the universal two-child policy was finally introduced in 2016, the families with an only daughter are more inclined to have another son [49]. Under such circumstances, female adolescents often face inequity in education and constrained by low expectations. Our study also confirms this conjecture to some extent. From the comparison of psycho-social characteristics between boys and girls (Table 2) we can see that proportion of male adolescents in rural areas was significantly higher than female adolescents (51.3 vs. 41.2%). In addition, significantly more girls have younger brother at home (33.8 vs. 20.0%) and less girls have older sister (11.9 vs. 27.5%). These unusual proportions could show that son preference is still widespread in China, especially in rural areas. Meanwhile, we observed that more female adolescents’ parents, both fathers and mothers, have higher education level. The role that fathers’ educational level plays in depressive symptoms may be explained by previous studies showing that fathers with a higher educational level tend to be warmer and more communicative with their children and to possess better emotional skills [53, 54]. Fathers with primary school education or below usually unable to provide favorable education and economic conditions to their daughters, which may also increase the prevalence of high depressive symptomatology. A study reported that father’s education level was positively associated with girl’s well-being in rural China [55], indicating that better educated fathers may value their daughters’ development more. Moreover, growing body of researches indicated that parental preference for sons might reduce under the modern outlook of fertility in urban areas but remained strong among rural-urban migrants [48, 49, 56], which means it’s the higher education levels of parents, not living areas, reduce the preference for sons.

### Strengths and limitations

To the best of our knowledge, this is the first large sample cross-sectional study to explore the different related factors of high depressive symptomatology in female and male adolescents in central China. The findings of this study provide etiological clues to gender difference in adolescent depression. At the same time, there are some limitations in this study. First, in this study, some variables are not included because they don’t have clear cause-and-effect relationship with depression or not objective enough, such as family financial situation, academic achievement, pressure of interpersonal relationship, etc. Second, our sample was confined to Hubei province, the conclusion needs further verification in other regions of China. Third, the CES-D scale was a brief instrument to measure the burden of depression, not able to provide a clinical depression diagnosis.

## Conclusion

Our study focuses on psycho-social factors related to female adolescents with high depressive symptomatology and gender differences in depressive symptoms among Chinese adolescents. We found that adolescents from single parent family, adolescents with older age and adolescents with less physical activity were more susceptible to high depressive symptomatology. However, fathers’ education background was only associated with high depressive symptomatology in female adolescents. Female adolescents whose father with primary school education or below deserves more attention. In China, especially in low educated communities, son preferences still widespread and popularization of rural compulsory education is the priority of China’s educational development. Furthermore, it is imperative to confirm these findings through nationally representative study.

## Data Availability

The de-identified database used in the current study are available from the corresponding author on reasonable request.

## Abbreviations

OR: Odds ratio
CI: Confidence interval
WHO: World Health Organization
CES-D: Center for Epidemiology Studies Depression Scale
PA: Physical activity
LBC: Left-behind children
IQR: Interquartile ranges
